# Conjoint measurement of disorder prevalence, test sensitivity, and test specificity: notes on Botella, Huang, and Suero's multinomial model

**DOI:** 10.3389/fpsyg.2013.00876

**Published:** 2013-11-22

**Authors:** Edgar Erdfelder, Morten Moshagen

**Affiliations:** Department of Psychology, University of MannheimMannheim, Germany

**Keywords:** multinomial modeling, validity, diagnostic accuracy, gold standard, imperfect reference

Botella et al. (2013) proposed two useful multinomial models for conjoint measurement of disorder prevalence rates in different populations (e.g., prevalence rates of dementia) and both the sensitivity and the specificity of the test used to assess this disorder (e.g., the Mini Mental State Examination, MMSE; Folstein et al., 1975). Their first model requires a perfect indicator of the disorder (i.e., a gold standard, GS), whereas the second model provides for indicators not perfectly correlated with the disorder (i.e., imperfect references, IR). In line with Lazarsfeld's (1950) latent-class model, the only requirement of the latter model is local stochastic independence of the IR and the test-based classification, that is, stochastic independence of the IR and the test result within subpopulations of individuals with vs. without the disorder.

The present comment addresses two shortcomings of the IR model and suggests ways to overcome them: (1) Lack of global identifiability in general and (2) lack of local identifiability when prevalence rates are homogenous across populations.

Problem (1). As acknowledged by Botella et al. (2013), the IR model is not globally identifiable. There are always two sets of sensitivity and specificity parameters for both the reference (*Se_R_* and *Sp_R_*, respectively) and the test (*Se_T_* and *Sp_T_*, respectively) that predict exactly the same outcome probabilities and therefore cannot be distinguished on grounds of model fit [see Botella et al. (2013), Table 1]. Despite the lack of uniqueness in parameter estimates, Botella et al. (2013) recommended use of the unconstrained IR model and to choose the set of parameter estimates that appears more plausible. However, besides introducing an unnecessary degree of subjectivity, a model that is consistent with parameter values incongruent with common sense is obviously too flexible and overly complex. For example, Botella et al.'s IR model allows for references and tests that are negatively correlated with the disorder under investigation, that is, for tools that measure the opposite of what they are supposed to measure. This is clearly not reasonable. In addition, their model lacks unique validity measures for both the reference and the test.

A simple way to remedy these problems is to constrain the sensitivity and specificity parameters in accordance with the two-high threshold model of detection (e.g., Snodgrass and Corwin, 1988; Waubert de Puiseau et al., 2012). In this refined model, the parameters of the IR model are reparameterized as follows:

(1) $$Se_{\text{R}}\;=D_{\text{R}}+(1-D_{\text{R}})\cdot B_{\text{R}}$$

(2) $$Sp_{\text{R}}\;=D_{\text{R}}+(1-D_{\text{R}})\cdot (1-B_{\text{R}})$$

The new parameters, *D_R_* and *B_R_*, denote validity and bias measures, respectively, for the IR [both in (0, 1)]. *D_R_* is the probability that the IR detects the true status (disorder present vs. absent), and *B_R_* represents the disorder-present bias (i.e., the probability of a positive diagnosis) given failure to detect the true status. Accordingly, the sensitivity and specificity parameter estimates of the test, *Se_T_* and *Sp_T_*, are reparameterized as functions of test validity and bias parameters *D_T_* and *B_T_*, respectively. Importantly, these reparameterizations jointly imply the order constraints *Se_R_* ≥ (1 − *Sp_R_*) and *Se_T_* ≥ (1 − *Sp_T_*)^1^ so that a positive diagnosis cannot be less likely given presence than given absence of the disorder. In other words, whereas the dimensionality of the parameter space remains unchanged (as the *Se* and *Sp* parameters are replaced by *D* and *B* parameters), the refined model restricts the admissible data space. As a consequence, in contrast to Botella et al.'s IR model, the refined model excludes negative correlations of the disorder with both the IR and the test. Moreover, introducing these order constraints renders the model globally identifiable (subject to the auxiliary condition of unequal prevalence rates, see below), thereby removing any ambiguity in interpretation.

As summarized in Table 1, fitting the refined model to the data sets analyzed by Botella et al. (2013, Table 2) results in the same goodness-of-fit statistics as observed for the original IR model^2^. This shows that the order constraints are perfectly in line with the data^3^. However, as a consequence of exclusion of negative correlations, model flexibility as measured by c_FIA_ is reduced for the refined model, resulting in better Minimum Description Length (MDL) indices of model fit than observed for the original IR model. An additional advantage of the refined model is that it provides unique validity and bias measures for both the reference and the test. For the MMSE data, for example, the test validity (0.736) is almost as large as the validity of the reference (0.876), although the difference in validities is statistically significant [ΔG^2^(1) = 8.60, *p* = 0.003]. Most importantly, unlike the original IR model, the refined model is globally identifiable so that there is only a single set of validity and bias estimates (and the corresponding sensitivity and specificity estimates) for both measurement tools involved (see Table 1).

**Table 1:** **Maximum likelihood parameter estimates, goodness-of-fit (*G*^2^), c_FIA_, and Minimum Description Length (MDL) measures for the original and the refined IR model applied to the AUDIT and the MMSE data of Botella et al. (2013, Table 2)**.

**Statistic/Estimate**	**AUDIT data**		**MMSE data**	
	**Original model**	**Refined model**	**Original model**	**Refined model**
*Se_R_*	0.996/0.000	(0.996)	0.876/0.000	(0.876)
*Sp_R_*	1.000/0.004	(1.000)	1.000/0.124	(1.000)
*D_R_*	–	1.000	–	0.876
*B_R_*	–	–	–	0.000
*Se_T_*	0.637/0.040	(0.637)	0.864/0.128	(0.864)
*Sp_T_*	0.960/0.363	(0.960)	0.872/0.136	(0.872)
*D_T_*	–	0.600	–	0.736
*B_T_*	–	0.098	–	0.486
*G*^2^(4)	13.99	13.99	12.14	12.14
*c_FIA_*	20.1	18.7	23.0	21.6
MDL	577.0	575.6	1493.6	1492.2

Parameter estimates in parentheses are derived from the corresponding validity and bias estimates using Equations (1) and (2). The two estimates for the original model correspond to the two maxima of the likelihood function. Note that the B_R_ parameter for the AUDIT data is not identifiable because D_R_ approaches the boundary of the parameter space.

Problem (2). To apply their models in situations where classification data are available from a single large study only, Botella et al. (2013) suggested a random split of this sample in *k* segments and to treat these segments as if they were drawn from *k* different populations. However, apart from sampling error, random splits necessarily result in the same prevalence rate in each of the random segments so that the same population classification matrix must hold for each data set. In effect, there are only 3 instead of 3*k* independent category probabilities available, implying that both the standard and the refined IR model (with *k* + 4 parameters each) cannot be identifiable. Hence, random splits of a large sample will be of no help. A possible remedy is to split the sample based on a third variable that has been observed in addition to the IR and the test result (say, gender, age group, profession, or religion), provided the assumption can be made that the prevalence rates, but not the sensitivity and specificity of the test and the reference, differ between the corresponding subpopulations. Unequal prevalences in at least two subpopulations suffice to ensure local identifiability. Thus, systematic splits of a single large sample may remedy the identifiability problem whereas random splits will not.

## Footnotes

^1^This follows from (1 − *Sp_R_*) = (1 − *D_R_*) · *B_R_* as implied by Equation (2) and, correspondingly, (1 − *Sp_T_*) = (1 − *D_T_*) · *B_T_*.

^2^The model specification and data files used to derive the results of Table 1 with multiTree (Moshagen, 2010) can be requested from the first author.

^3^Although not required for the present data, it is also possible to conduct a formal ΔG^2^ difference test of the refined model against the original IR model. However, because both models include the same number of parameters and differ by a parametric order constraint only, the asymptotic distribution under the null hypothesis is a mixture of χ^2^ distributions (Iverson, 2006) rather than a standard χ^2^ distribution. The parametric bootstrap as implemented in multiTree (Moshagen, 2010) can be used to approximate this distribution.
